# Redo TEP in recurrent inguinal hernia After TEP/TAPP: Outcomes and feasibility

**DOI:** 10.1007/s10029-026-03646-2

**Published:** 2026-03-16

**Authors:** Navid Tabriz, Dimitri Khmara, Dirk Weyhe

**Affiliations:** University Hospital for Visceral Surgery, Pius-Hospital, Oldenburg, Germany

**Keywords:** Re-TEP, Recurrent inguinal hernia, Laparoendoscopic hernia repair

## Abstract

**Purpose:**

The optimal management of recurrent inguinal hernia following previous laparoendoscopic repair lacks robust scientific evidence and varies across guidelines. Due to a paucity of data, the European Hernia Society currently recommends open anterior repair for the management of recurrent inguinal hernias after a previous laparoendoscopic repair, based solely on expert opinion. However, repeat endoscopic repair can yield favorable outcomes in experienced hands. This study aimed to compare patient outcomes between repeat endoscopic repair (ReTEP) and the Lichtenstein technique for recurrent hernias after initial TAPP or TEP.

**Methods:**

Adult patients undergoing surgery for first recurrence after laparoendoscopic repair were included. Intra- and postoperative morbidity was analyzed retrospectively, and symptoms and quality of life were assessed prospectively using clinical and ultrasound examination, the Carolinas Comfort Scale (CCS), and the COMI (Core Outcome Measurement Index)-Hernia questionnaire.

**Results:**

Between January 2012 and March 2020, the center performed 48 ReTEPs and 45 Lichtenstein hernioplasties for the first recurrence of inguinal hernia after primary endoscopic surgery. Both groups were generally comparable in terms of age, BMI and intrinsic perioperative risk factors. The rate of conversion from ReTEP to Lichtenstein procedure was 27,3%, remained consistent over the years and showed no correlation with surgeon’s expertise. There were no statistically significant differences in the frequency and severity of complications between ReTEP and Lichtenstein. The Lichtenstein procedure was significantly superior in the categories “foreign body sensation” and “pain” assessed using the CCS and the second recurrencies were more frequently observed after ReTEP.

**Conclusion:**

The findings support the expert suggestion of HerniaSurge group regarding the change of procedure for managing recurrent inguinal hernia following initial endoscopic surgery. In this case the Lichtenstein operation should be considered.

## Introduction

Each year, approximately 20 million inguinal hernia repairs are performed worldwide, including about 275,000 in Germany. The lifetime risk of developing an inguinal hernia is roughly 27% for men and about 3% for women [1, 2]. Inguinal hernia repair is therefore among the most frequently performed surgical procedures. Despite advances in surgical techniques toward laparoendoscopic approaches, these techniques are not free from recurrence. According to the German Hernia Registry, about 11% of inguinal hernia repairs are carried out for recurrent hernias. The incidence of reoperation for recurrence within the first year is approximately 0.95%, and the estimated recurrence rate at 10-year follow-up is about 5.01% [3, 4].

While guideline-based recommendations (evidence grade I) exist for the operative technique in primary inguinal hernias and for repair of recurrences after a prior open operation [5], the optimal management of recurrent inguinal hernia following a laparoendoscopic primary repair remains unclear. The International Endohernia Society (IEHS) guideline rates repeated laparoscopic repair for recurrence after TAPP or TEP (reTAPP) as “feasible for experienced endoscopic surgeons” and considers it superior to repeated extraperitoneal repair (reTEP) (evidence level 5) [6, 7]. In contrast, the European Hernia Society (EHS) guideline states that, in the event of a recurrence after laparoendoscopic repair, a change to an open technique offers clear advantages because the procedure is performed in an undisturbed tissue plane (evidence level 4) [8, 9].

The international guideline for the treatment of inguinal hernia published in 2018 by the HerniaSurge Group (currently valid) recommends a complementary strategy [5] (i.e., switching the surgical approach—open repair after laparoendoscopic primary repair and vice versa) for recurrences after TAPP or TEP, even though the accompanying commentary did not present improved evidence and referred to the above mentioned 2009 EHS recommendation, which, as noted, carries a low evidence grade. On the other hand, in the same year, a position statement of the German Hernia Society (DHG) addressed the key HerniaSurge recommendations. With regard to complementary treatment of recurrent hernias, this statement judged the international literature to be unambiguous and strongly recommended adhering to the complementary approach, with any exceptions being explained to the patient [10].

In summary, both European and international recommendations for reoperation after an initial TAPP or TEP largely reflect expert opinion and are supported by low-quality evidence. There are neither systematic reviews nor randomized controlled trials addressing the optimal management of recurrent inguinal hernias after laparoendoscopic repair. The role of ReTEP has not been sufficiently investigated, particularly with respect to perioperative morbidity and postoperative quality of life.

Accordingly, this study hypothesized that, in the surgical treatment of recurrence after laparoendoscopic primary inguinal hernia repair, operative outcomes of ReTEP do not differ from those of the complementary open Lichtenstein procedure with regard to patient safety and quality of life.

The primary endpoint of this investigation was to compare intra- and postoperative morbidity of ReTEP with that of the Lichtenstein operation for the first recurrence after a laparoendoscopic primary procedure, and to compare ReTEP with primary TEP in a recurrence after an initial open operation. The secondary endpoint was the assessment of postoperative quality of life after ReTEP compared with the complementary Lichtenstein repair.

## Materials and methods

This study was designed, conducted, and reported in accordance with the STROBE statement for observational studies.

**Fig. 1:**
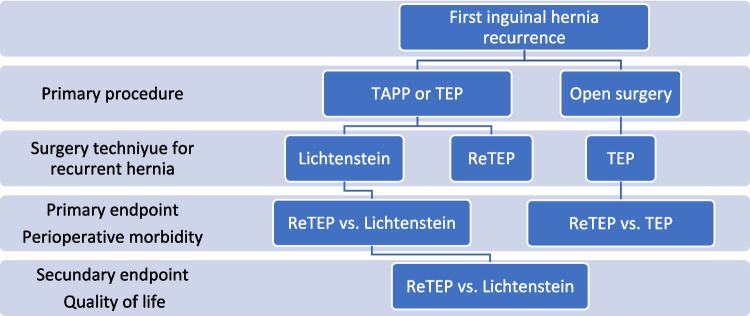
Study design. Graphical overview of cohort assembly and analysis

**Fig. 2:**
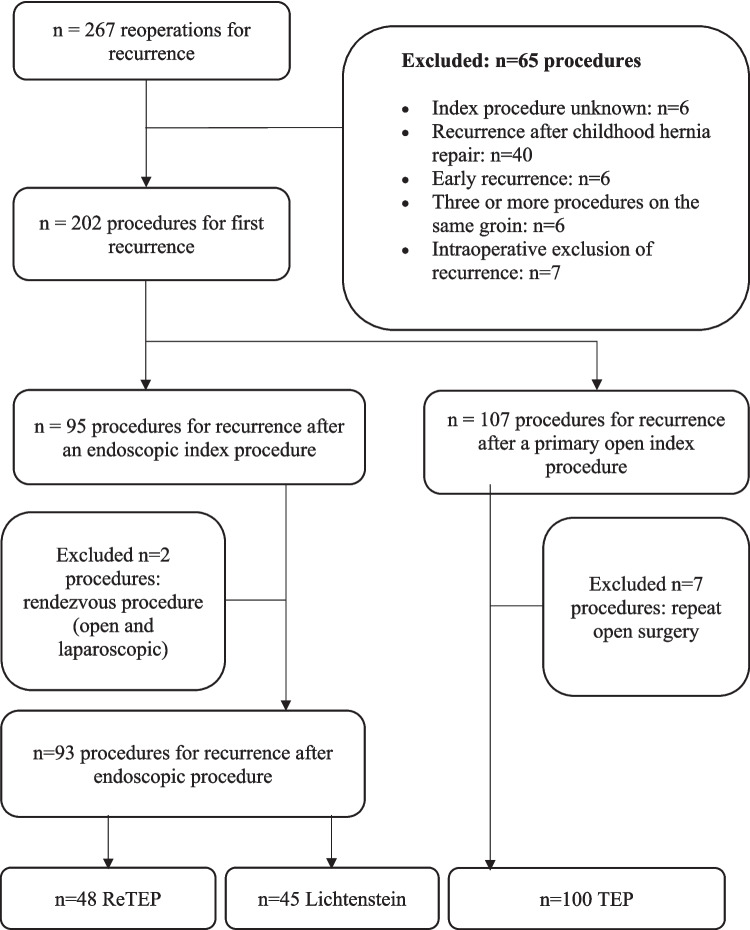
Flow diagram of inclusion and exclusion. From 267 reoperations, 65 exclusions left 202 first-recurrence procedures. Among 95 recurrences after endoscopic index repair, two hybrids were excluded, leaving ReTEP (n = 48) and Lichtenstein (n = 45). Among 107 recurrences after prior open repair, TEP (n = 100) remained

A single-center observational study based on prospectively captured clinical data from the Department of Visceral Surgery, Pius-Hospital Oldenburg (University Hospital for Visceral Surgery, Oldenburg, Germany), covering the period January 2012 to March 2020 was performed. Cases were identified within the Herniamed quality registry and cross-verified against the institutional hospital information system. The study protocol was approved by the Medical Ethics Committee of the Carl von Ossietzky University Oldenburg (reference 2019–122). Figs. 1 and 2

### Eligibility criteria

We included adult patients (female and male, ≥ 18 years) who underwent either (i) repeat total extraperitoneal repair (reTEP) or (ii) complementary open Lichtenstein repair for a first ipsilateral inguinal hernia recurrence after an initial laparoendoscopic primary repair (TEP or TAPP). For completeness of benchmarking and as a comparison group, we also included patients who underwent a TEP after a primary open repair during the same period. After eligibility screening, cases were assigned to three analytic groups:

ReTEP (repeat total extraperitoneal repair after primary TEP/TAPP),

Lichtenstein (open repair after primary TEP/TAPP),

TEP (TEP after a primary open repair).

### Demographic and baseline variables

From medical records and case documentation (including clinic letters, preoperative assessment forms, operative notes, ward rounds, discharge summaries, and post-discharge entries), we extracted: date of birth, sex, age at recurrence surgery, height, weight, body mass index (BMI), patient-related risk factors for perioperative complications, and the American Society of Anesthesiologists Physical Status (ASA-PS) class.

Patient-related risk factors were categorized analogously to the Herniamed registry to facilitate comparability, and encompassed comorbid conditions and medications/substances associated with elevated risks of bleeding, infection, or recurrence. A positive bleeding history, continued use of antiplatelet agents or oral anticoagulants, and perioperative bridging with low-molecular-weight or unfractionated heparin were classified as bleeding-risk factors. Infectious risk was deemed higher with known congenital, acquired, or drug-induced immunosuppression, diabetes mellitus, obesity, or tobacco use. Our institutional practice includes routine perioperative antibiotic prophylaxis for recurrence repair; therefore, antibiotic use was not captured as a variable. Factors associated with increased recurrence risk included COPD, ASA class III–IV, obesity, and tobacco use.

### Operative data

For the index (primary) operation we recorded: date and duration, EHS classification and defect size, operative approach, and mesh size.

For the recurrence operation we recorded: admission and discharge dates, length of stay, date and duration of surgery, preoperative pain level, hernia type and EHS defect size, conversion (yes/no), intraoperative findings (e.g., hernia, cord lipoma, preperitoneal adhesions, undersized or malpositioned prior mesh), operative technique, mesh size and fixation method, drain placement, surgeon seniority (resident under supervision, board-certified attending, senior attending/consultant, or department chair), intra- and postoperative complications, and postoperative pain. Cumulative length of hospital stay was recorded.

Intraoperative complication was defined as any event deviating from the planned course requiring corrective action (e.g., organ injury requiring repair, bleeding with loss of visualization necessitating conversion). Consequence-free peritoneal nicks, pragmatic neurectomy, or prophylactic ligation and transection of a vessel were not counted as intraoperative complications.

Postoperative complication encompassed any deviation from an uncomplicated course within 90 days, including hematoma, seroma, surgical-site infection or wound-healing disturbance, early recurrence, and other events (e.g., pneumonia, urinary retention, postoperative delirium, cardioembolic events). Complications were documented and graded according to the Clavien–Dindo classification. We examined the clinical impact of each complication on recovery and compared rates against national Herniamed benchmark data.

Given the heterogeneous morbidity reported for repeat laparoendoscopic repair in the literature, we additionally assembled a reference cohort undergoing TEP after prior open repair to benchmark morbidity against ReTEP after TEP/TAPP.

Conversion rate from planned ReTEP to open repair served as a key feasibility metric. We identified all cases where extraperitoneal re-exploration was indicated and initiated; the conversion rate was calculated as conversions divided by all attempted extraperitoneal reoperations. Reasons for conversion (e.g., inadequate working space, unmanageable intraoperative complication) and temporal distribution were analyzed to assess potential learning-curve effects.

We also screened for technical factors predisposing to recurrence (e.g., cranial mesh displacement, retained cord lipoma or missed hernia, undersized mesh, inadequate dissection space due to adhesions).

### Prospective quality of life assessment and follow-up

The prospective recruitment commenced on 01 July 2020. All patients in the ReTEP and Lichtenstein groups were contacted in writing and informed about the study aims. Patients were invited to schedule an in-person follow-up visit by telephone.

Follow-up comprised validated QoL questionnaires—the Carolinas Comfort Scale (CCS) and the Core Outcome Measurement Index for hernia (COMI-hernia)—plus a standardized clinical examination and surgeon-performed inguinal ultrasound.

Follow-up visits took place in the surgical outpatient clinic at Pius-Hospital Oldenburg. A structured interview (study-specific form) captured postoperative course and current symptoms. Physical examination focused on detecting recurrence and surgery-related complications; potential functional disturbances (e.g., micturition or sexual dysfunction, weather sensitivity) were recorded.

Although the role of ultrasound in inguinal hernia follow-up is debated, we considered it essential for objective documentation and communication of findings. Ultrasound assessed for seroma, meshoma (chronic mesh shrinkage without fistula), or recurrent hernia. QoL data were merged with clinical records, Herniamed entries, and follow-up forms in a spreadsheet for analysis.

### Quality of life instruments

The CCS is a validated, sensitive, hernia-specific instrument assessing mesh awareness (foreign-body sensation), pain, and movement limitation during common activities (lying, bending forward, standing up, daily activities such as washing/dressing, coughing/deep breathing, walking, stair climbing, and sports). It contains 23 items rated on a 0–5 Likert scale; item scores are summed (range 0–115), with higher totals indicating worse QoL. Because total-score comparisons are more informative for pre-/postoperative change than cross-sectional cohort contrasts, we dichotomized responses for between-group analysis: any item ≥ 2 classified the patient as “symptomatic”; items 0–1 were “asymptomatic.” The proportion symptomatic was calculated per cohort.

The COMI-hernia is a multidimensional long-term outcome instrument covering pain, function, well-being, health-related QoL, social limitation, work incapacity, global treatment success, satisfaction, and complications. Responses are scored 0–5; each domain is transformed to 0–10 by subtracting 1 and multiplying by 2.5: (score − 1) × 2.5 (e.g., raw 3 → 5; raw 5 → 10). Transformed domain scores are summed with the numeric rating scale pain score (0–10) and averaged to yield the COMI Total Score (lower is better).

### Impact of the COVID-19 pandemic

Recruitment for prospective follow-up was severely constrained by pandemic-related hygiene measures and reduced response rates, limiting in-person visits by asymptomatic individuals. A renewed recruitment effort was launched in early 2022, using postal and telephone contacts. When onsite assessment was not possible or not desired, QoL was collected via mailed and telephone interviews. The study was closed on 01 July 2022.

### Data protection and confidentiality

The study was conducted in accordance with the Declaration of Helsinki and Good Clinical Practice. Data processing complied with German data protection law. Patients consented to registry-based data use; study analyses were performed on linked, pseudonymized datasets with restricted re-identification. The final dataset was anonymized, stored for 10 years, and approved by the institutional data protection officer.

### Statistical analysis

After data lock, all variables were imported into SPSS for analysis. We first described each study group using demographic characteristics (age, sex, height, weight, BMI), baseline morbidity (ASA-PS, prior operations in the groin region), and risk factors for perioperative complications. Operative variables (duration, urgency, preoperative pain, hernia location/type, EHS defect size, conversion, intraoperative findings, procedure performed, mesh size and fixation, drain placement, surgeon seniority), complications (intra- and postoperative), postoperative pain, length of stay, and QoL metrics (COMI-hernia Total Score, CCS) were summarized descriptively.

We report absolute and relative frequencies for categorical variables; for continuous variables, we present measures of central tendency (minimum, maximum, mean, median) and dispersion (standard deviation). Distributions of quantitative variables were inspected using histograms. For normally distributed metric data, we calculated means and standard deviations. Group comparisons for nominal and ordinal endpoints used Pearson’s chi-square test. Ordinal or non-normally distributed variables were compared using the Mann–Whitney U test or Kruskal–Wallis H test, as appropriate. Two-sided p-values < 0.05 were considered statistically significant.

## Results

A database query identified 267 reoperative procedures for inguinal hernia recurrence from January 2012 to March 2020. After exclusions (unknown index procedure, childhood repair, early recurrence, ≥ 3 procedures ipsilaterally, or intraoperative exclusion of recurrence), 202 first-recurrence procedures remained. Of these, 95 followed a prior laparoendoscopic repair and were allocated to ReTEP (n = 48) or Lichtenstein (n = 45) after excluding two hybrid procedures. Separately, 100 TEPs after prior open repair were analyzed for benchmarking.

### Group characteristics

The respective group characteristics are provided in the Tables 1 and 2. Overall, no statistically significant differences were observed between the groups concerning age, BMI, priory surgeries, risk for bleeding, infection or recurrence and ASA classification. Thus, the groups were comparable.

**Table 1:** Descriptive characteristics: ReTEP vs. Lichtenstein

Variable	ReTEP	Lichtenstein	p-value
Age, years (mean ± SD)	63.9 ± 10.1	65.2 ± 13.8	0.193
BMI, kg/m^2^ (mean ± SD)	27.0 ± 3.2	26.8 ± 3.3	0.674
Prior lower-abdominal surgery, %	16.7	26.7	0.241
Bleeding-risk, %	14.6	31.1	0.057
Infection-risk, %	29.2	35.6	0.879
Recurrence-risk, %	43.8	57.8	0.924
ASA I/II/III, %	22.9/58.3/18.8	11.1/57.8/31.1	0.073

**Table 2:** Descriptive characteristics: ReTEP vs. TEP

Variable	ReTEP	TEP	p-value
Age, years (mean ± SD)	63.9 ± 10.1	62.1 ± 15.4	0.242
BMI, kg/m^2^ (mean ± SD)	27.0 ± 3.2	26.5 ± 3.2	0.596
Prior lower-abdominal surgery, %	16.7	19.0	0.731
Bleeding-risk, %	14.6	21.0	0.351
Infection-risk, %	29.2	37.0	0.750
Recurrence-risk, %	43.8	41.0	0.275
ASA I/II/III, %	22.9/58.3/18.8	15/66/19	0.446

### Procedure-related data

Table 3 presents the operative and perioperative findings of the three comparison groups. Notably for postoperative pain, Mann–Whitney U test showed significantly lower pain after ReTEP vs. Lichtenstein (p = 0.01); no significant differences for other pairs.

**Table 3:** Intra- and perioperative data of the Three Patient Groups

Variable	ReTEP (n = 48)	Lichtenstein (n = 45)	TEP (n = 100)
Interval since index operation, years (mean ± SD)	7.4 ± 5.2	3.7 ± 3.8	14.6 ± 10.1
Surgeon expertise, n (%)			
Residents	0 (0%)	3 (6.7%)	6 (6%)
Specialists	4 (8.3%)	8 (17.8%)	15 (15%)
Senior attendings	34 (70.8%)	28 (62.2%)	63 (63%)
Department chair	10 (20.8%)	6 (13.3%)	16 (16%)
Side, right/left	25/23	22/23	60/40
Cord lipoma, n (%)	6 (12.5%)	6 (13.3%)	15 (15%)
Adhesions, n (%)	36 (75%)	21 (46.7%)	19 (19%)
EHS classification, n (%)			
Medial	25 (52.1%)	16 (35.6%)	24 (24%)
Lateral	22 (45.8%)	25 (55.6%)	60 (60%)
Femoral	0 (0%)	1 (2.2%)	0 (0%)
Combined	1 (2.1%)	3 (6.7%)	16 (16%)
Defect size (EHS), n (%)			
EHS 1	4/40 (10%)	6/36 (16.7%)	19/96 (19.8%)
EHS 2	26/40 (65%)	16/36 (44.4%)	59/96 (61.4%)
EHS 3	10/40 (25%)	14/36 (38.9%)	18/96 (18.8%)
Mesh dimensions (mean ± SD)			
Length, cm	13.7 ± 1.1	11.9 ± 1.8	13.7 ± 1.0
Width, cm	9.6 ± 1.5	8.2 ± 1.6	9.2 ± 0.8
Drains, n	17	14	16
Operative time, min (mean ± SD)	81 ± 35	83 ± 30	49 ± 22
Postoperative VAS ≤ 3, n (%)	40/43 (93%)	35/41 (85.4%)	92/97 (94.8%)
Length of stay, days (mean ± SD)	2.0 ± 0.8	2.5 ± 1.6	2.0 ± 1.7

### Conversions from ReTEP/TEP to Lichtenstein

Eighteen procedures initiated endoscopically were converted to Lichtenstein, all due to adhesions preventing safe working-space creation. Annual numbers showed no decline over time. Conversion by operator level (relative to completed ReTEP): specialists 42.8% (3/7), senior attendings 22.7% (10/44), chair 33.3% (5/15) showed no significant difference (Kruskal–Wallis p = 0.476). Overall ReTEP conversion rate was 27.3% (18/66). In contrast, only 2/102 (2%) complementary TEPs converted to open (p < 0.001). Documented adhesions were significantly associated with conversion (p < 0.001).

Across the ReTEP and Lichtenstein cohorts, intraoperative courses were largely uneventful with no organ or nerve injuries in either group. In the ReTEP arm, two minor peritoneal nicks occurred without sequelae; epigastric vessels were prophylactically clipped in two cases and three controllable epigastric bleeds were recorded. In the Lichtenstein arm, one controllable epigastric bleed occurred during a case converted from endoscopic exploration due to adhesions; pragmatic neurectomy was performed in five patients as a preventive measure against chronic pain.

Postoperatively, the most common event was hematoma formation and was predominantly self-limiting in both groups. In ReTEP, hematoma occurred in 33.3% (16/48), with one ambulatory evacuation and one suspected infected hematoma managed by aspiration plus empiric antibiotics; seroma 4.2% (2/48), one minor wound-healing disturbance, and a single transient urinary retention were documented. Overall, 62.5% (30/48) of ReTEP patients had an uncomplicated course; 33.3% experienced Clavien–Dindo grade 1 events; two patients had combined events (one Clavien-Dindo G1 + G1, one G1 + G3a).

In the Lichtenstein cohort, hematoma 35.6% (16/45; two operative evacuations), seroma 6.7%, wound-healing disorders 6.7% (one secondary closure), one superficial SSI, no mesh infections, one transient delirium, and one case of metamizole-induced leukothrombocytopenia were reported. 53.3% (24/45) had an uneventful course; Clavien–Dindo distribution was Grade 1 33.3%, Grade 2 4.4%, Grade 3 8.7%. Comparing ReTEP vs Lichtenstein, there was no significant difference in the primary endpoint of perioperative morbidity (all p > 0.05; see Table 4).

**Table 4:** Complication profiles: ReTEP vs. Lichtenstein. No significant difference for the primary endpoint (perioperative morbidity): p > 0.05

	ReTEP (n = 48)		Lichtenstein (n = 45)		Chi-square (p-value)
	n	%	n	%	
Intraoperative complications	3	6.3	1	2.2	0.339
- Bleeding	3	6.3	1	2.2	0.339
- Organ injury	0	0	0	0	—
- Nerve injury	0	0	0	0	—
Postoperative complications	21	43.8	27	60.0	0.806
- Hematoma	16	33.3	16	35.6	0.822
- Seroma	2	4.2	3	6.7	0.593
- Wound-healing disorder	1	2.1	3	6.7	0.276
- Surgical-site infection	1	2.1	1	2.2	0.963
- Other	1	2.1	4	8.8	0.146
Patients without complications	30	62.5	24	53.3	0.261
Clavien–Dindo					
- Grade 1	16	33.3	15	33.3	1.000
- Grade 2	0	0	2	4.4	0.140
- Grade 3	2	4.2	4	8.7	0.354
- Grade 4	0	0	0	0	—
- Grade 5	0	0	0	0	—

A comparison of intra- and postoperative adverse events between the cohorts is summarized in Table 4. No significant difference was found for the primary endpoint, perioperative morbidity (p > 0.05).

In the TEP cohort (recurrence after primary open repair), six intraoperative bleeding events (two focal, four diffuse) were managed without organ/nerve injury. Postoperatively, hematoma 12% (two aspirations; one suspected infection treated conservatively) and one life-threatening rebleed required two reoperations and ICU care (Clavien–Dindo 4a). Seroma 6% (two aspirated) and several “other” events (e.g., ileus due to preperitoneal bowel prolapse requiring surgery; delirium; urinary retention; constipation) were recorded. 73% had an uncomplicated course. Versus ReTEP, TEP showed a significantly lower rate of postoperative hematoma (16.0% vs 33.3%; p = 0.016; RR 2.08 for ReTEP), while all other comparisons were non-significant; preoperative bleeding-risk profiles did not differ (p = 0.351; see Table 5).

**Table 5:** Complication profiles: ReTEP vs. TEP; Postoperative hematoma significantly less frequent after TEP (p = 0.016); RR for hematoma (reTEP vs. TEP) = 2.08. All other comparisons not significant (p > 0.05). Cohorts comparable for preoperative bleeding-risk factors (p = 0.351)

	ReTEP (n = 48)		TEP (n = 100)		Chi-square (p-value)
	n	%	n	%	
Intraoperative complications	3	6.3	6	6.0	0.952
- Bleeding	3	6.3	6	6.0	0.952
- Organ injury	0	0	0	0	—
- Nerve injury	0	0	0	0	—
Postoperative complications	21	43.8	29	29.0	0.123
- Hematoma	16	33.3	16	16.0	0.016
- Seroma	2	4.2	6	6.0	0.644
- Wound-healing disorder	1	2.1	0	0	0.148
- Surgical-site infection	1	2.1	1	1.0	0.593
- Other	1	2.1	6	6.0	0.216
Patients without complications	30	62.5	73	73.0	0.194
Clavien–Dindo					
- Grade 1	16	33.3	19	19.0	0.055
- Grade 2	0	0	4	4.0	0.160
- Grade 3	2	4.2	3	3.0	0.713
- Grade 4	0	0	1	1.0	0.487
- Grade 5	0	0	0	0	—

### Prospective quality of life data

**Fig. 3:**
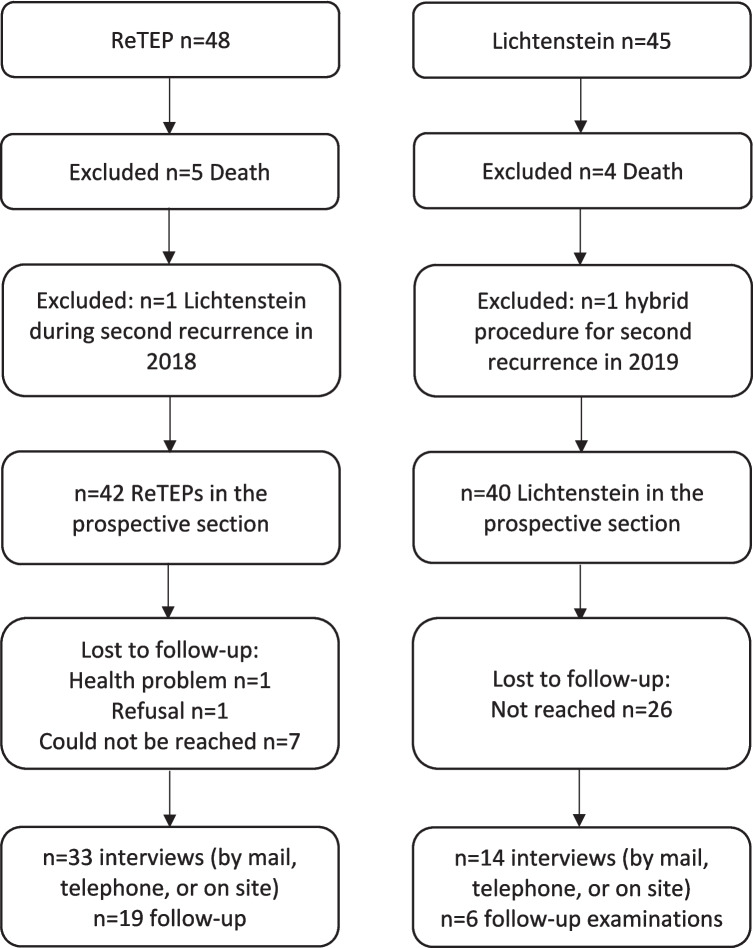
Flow of the prospective study phase

To assess the study’s secondary endpoint, we prospectively recorded quality of life (QoL) and conducted follow-up examinations in the ReTEP and Lichtenstein cohorts. The flow of the prospective phase is shown in Fig. 3.

Five ReTEP and four Lichtenstein cases were excluded because the patients had died by the time of assessment. In each arm, one additional patient had already undergone a second-recurrence repair with another technique before study start and was therefore excluded from invitations. Ultimately, invitations were sent to 42 ReTEP and 40 Lichtenstein patients.

Because of COVID-19, interviews were completed in 33/42 ReTEP patients (78.6%); in-person follow-up was performed in 19/42 (45.2%). In the Lichtenstein cohort, QoL data were obtained for 14/40 patients (35%); 6/40 (15%) attended in person. In the ReTEP cohort, one patient could not participate due to a severe health issue and one declined. Eight patients after laparoendoscopic repair and 26 after open repair could not be reached by mail/phone. Data collection ended three months after the second recruitment wave began.

Follow-up duration in the ReTEP group ranged 5–105 months (mean 37 ± 27). In the Lichtenstein group, mean follow-up was 46 ± 29 months (range 25–98).

Using the Carolinas Comfort Scale (CCS), the share of symptomatic patients was calculated. After ReTEP, 9/33 (27.3%) reported foreign-body sensation, 5/33 (15.1%) movement limitation, and 9/33 (27.3%) relevant pain; overall, 12/33 (36.4%) were symptomatic in at least one domain. In the Lichtenstein cohort, no patient met symptomatic criteria on CCS. Chi-square testing showed significant between-group differences for foreign-body sensation, pain, and for the overall proportion of symptomatic patients (see Table 6).

**Table 6:** Results of the prospective assessment

	ReTEP (n = 33)		Lichtenstein (n = 14)		p-value
	n	%	n	%	
Symptomatic per CCS (any domain)	12	36.4	0	0	0.009
Foreign-body sensation	9	27.3	0	0	0.03
Movement limitation	5	15.1	0	0	0.123
Pain	9	27.3	0	0	0.03
COMI-hernia Total Score (mean ± SD)	—	1.4 ± 0.3	—	0.8 ± 0.2	0.334
Hernia recurrence	6/19	31.6	1/6	16.7	0.478

The COMI-hernia Total Score was low in both groups (ReTEP mean 1.4 ± 0.3; range 0–6.6; Lichtenstein mean 0.8 ± 0.2; range 0–2.4) with no significant difference (Mann–Whitney U, p = 0.334).

At in-person follow-up after ReTEP, 6/19 (31.6%) reported pain (rest pain 15.8%; pressure pain 21.1%), and 5/19 (26.3%) reported foreign-body sensation. At the time of examination, 10/19 (52.6%) felt not impaired, 5/19 (26.3%) slightly impaired, and 3/19 (15.8%) moderately impaired; one patient reported very strong symptom burden. Overall satisfaction ranged from very satisfied 11/19 (57.9%), moderate 4/19 (21.1%) to low 4/19 (21.1%). Clinical exam suggested hernia recurrence in 5/19 ReTEP patients; ultrasound confirmed recurrence in all five. No seroma were detected; no weather sensitivity or voiding disorders were observed; one patient reported erectile dysfunction.

Among six examined Lichtenstein patients, one reported intermittent groin pain (3–5 times per week) with mild impairment; two were moderately satisfied and the remainder highly satisfied. One patient reported weather sensitivity. Clinical and ultrasound examinations were unremarkable; no recurrence was detected.

Exclusions due to death; additional exclusions for prior second-recurrence repair; invitations sent; completed interviews and in-person visits; losses to follow-up during COVID-19.

Despite pandemic-limited follow-up, CCS outcomes favored Lichtenstein (0% symptomatic) over ReTEP (36.4% symptomatic in any domain), with significant differences for foreign-body sensation and pain. Global QoL by COMI-hernia was similarly low in both groups (no significant difference). Sonographic follow-up showed recurrences after ReTEP (≥ 12.5%) and none detected in examined Lichtenstein patients; overall recurrence proportions trended higher after ReTEP but were not statistically different (p = 0.06).

### Analysis of second recurrences

Second inguinal hernia recurrences were identified in 5/19 ReTEP patients at follow-up Management ranged from watchful waiting to complementary open repair. Including one previously known and operated recurrence (excluded from the prospective phase), the cumulative recurrence rate after ReTEP was at least 31.6% (6/19) In the Lichtenstein cohort, no recurrence was detected at follow-up; together with one recurrence identified retrospectively, the cumulative rate was at least 16.7% (1/6). A chi-square comparison yielded p = 0.478, i.e., no statistically significant difference between cohorts.

## Discussion

The optimal surgical strategy for recurrent inguinal hernia after a prior laparoendoscopic index repair remains unsettled. Current guideline recommendations largely reflect expert opinion and are constrained by limited, heterogeneous evidence—predominantly retrospective designs, small cohorts, and inconsistent outcome definitions. In particular, the role and risk–benefit profile of a repeat posterior approach (redo total extraperitoneal repair, ReTEP) has not been adequately delineated against the more traditional complementary anterior Lichtenstein repair.

This study therefore compared perioperative morbidity and patient-reported outcomes (PROs) between ReTEP and Lichtenstein in first recurrences after TEP/TAPP, and used a TEP cohort as a benchmark to contextualize morbidity in a virgin versus a re-entered posterior plane.

### Perioperative morbidity

In our center’s experience, overall intra- and postoperative complication rates and their Clavien–Dindo severities did not differ significantly between ReTEP and Lichtenstein. Absolute postoperative complication proportions in both arms (≈44% for ReTEP; ≈60% for Lichtenstein) appeared higher than those reported in analyses of the German Herniamed registry [11]. This disparity is best explained by definition and capture effects rather than true excess morbidity: we prospectively counted all bleeding events and all postoperative deviations (Clavien–Dindo grades 1–5) to avoid selection bias.

Registry publications likely under-ascertain minor hematomas/bleeds, effectively counting mostly revision-requiring events (≥ grade 3). When our analysis is limited to revision-requiring postoperative bleeds, the rate (≈2.1%) aligns with national estimates.

The Danish registry analysis, which more comprehensively captured events after laparoendoscopic re-repair, found postoperative complications in 45% of cases—very close to our ReTEP —reinforcing that methodological rigor and definitions drive apparent differences across datasets [12]. On the other hand, a single-center study has previously demonstrated that in 30 ReTEP patients, the procedure was successfully performed in 100% of cases, without increased perioperative morbidity and with a quality of life comparable to that of patients undergoing primary TEP [13]. This finding is somewhat surprising and contrasts with both our results and the existing literature. However, perioperative complications were not recorded in the same level of detail as in our study.

The broader literature echoes this heterogeneity: reported postoperative complication rates after endoscopic re-repair range from 0 to 50% [14–21], reflecting divergent definitions (what constitutes a “complication”), ascertainment windows, and follow-up intensity. Consequently, direct cross-study comparisons are unreliable unless outcome taxonomies are standardized.

### ReTEP versus TEP—benchmarking the posterior plane

The benchmark cohort—complementary TEP after a primary open index repair—clarifies what morbidity is attributable to re-entering versus first-entering the posterior space. In our data, postoperative hematoma occurred significantly more often after ReTEP than after TEP (≈33% vs ≈16%; p≈0.016), despite similar distributions of bleeding-risk factors. Most events were Clavien-Dindo Grade 1 (self-limiting), but this might be clinically relevant for patient counseling.

Mechanistically, the difference aligns with operative findings: preperitoneal adhesions were far more common in ReTEP than in TEP, reflecting fibrotic, distorted planes after a prior posterior repair. Dissection in a scarred preperitoneal field plausibly increases bleed risk and challenges hemostasis. This observation supports the long-standing expert recommendation to switch planes (posterior ↔ anterior) for recurrent hernia, particularly when the previously operated plane is expected to be hostile [8, 10].

### Feasibility and conversions

Conversion from intended ReTEP to open anterior repair was non-trivial (≈27%), driven almost exclusively by dense preperitoneal adhesions that precluded safe working-space creation. Conversion did not vary significantly by surgeon seniority and did not decline over time, arguing for an intrinsic difficulty of re-entering the posterior plane rather than a simple learning-curve effect. In contrast, complementary TEP after a primary open index showed a very low conversion rate (≈2%), again highlighting the differencebetween virgin and operated posterior planes.

Published conversion rates are likewise heterogeneous (0–24%) and blend two distinct redo approaches—ReTAPP [15, 16, 18, 19, 21, 22] and ReTEP [13, 17]. Some series report very low conversion with ReTAPP, which may reflect easier access to the preperitoneal space via the peritoneal cavity. Our conversion rate closely mirrors prior ReTEP-specific reports and reinforces that redo extraperitoneal repair is technically demanding. In practice, centers pursuing ReTEP should anticipate a material conversion contingency and counsel patients accordingly.

### Patient-reported outcomes

The secondary endpoint—quality of life—demonstrated clinically and statistically meaningful differences between strategies on the Carolinas Comfort Scale (CCS):

After ReTEP, 36.4% reported symptoms (pain or foreign-body sensation: 27.3% each), whereas no symptoms occurred after Lichtenstein, with significant between-group differences.In contrast, the COMI-hernia total score—a multidimensional global QoL index—was low in both arms with no significant difference, suggesting that while mesh-related or site-specific symptoms are more common after ReTEP (as captured by CCS), overall function and life quality remain generally favorable with either approach. In Öberg et al. [23], pain-related functional limitation was reported by 21% of patients after both laparoendoscopic and open repair of recurrent inguinal hernia, with no statistically significant difference between groups (27/131 vs. 85/406; p = 0.94). The proportion of symptomatic patients by the Carolinas Comfort Scale (CCS) was 7% overall, again without between-group difference (8/121 vs. 26/373; p = 0.89).

Two caveats temper interpretation: first, COVID-19 constrained recruitment and reduced in-person evaluation, risking non-response bias. Second, the Lichtenstein PRO sample was small, and a 0% symptomatic rate is likely an underestimate of true symptom prevalence. Even so, the direction and magnitude of the CCS differences favor the complementary anterior approach after a prior posterior repair.

Possible mechanisms for higher CCS symptomatology after ReTEP could be that the endoscopic redo strategy typically leaves the prior mesh in situ and adds a second mesh in the same compartment. This layering of foreign material can create a less compliant, multi-laminar augmentation with overlapping scar plates, perceived as foreign-body sensation. Additionally, reconstitution of the deep ring with a ventral/caudal mesh edge near the cord structures (or round ligament/ovarian vessels) raises the risk of genitofemoral nerve irritation, particularly if the maneuver is repeated in a scarred field—plausibly increasing chronic pain potential. Comparative studies quantifying mesh load and anatomical interfaces in re-repair are lacking and merit prospective evaluation.

### Second recurrences—direction of effect and detection issues

Second-recurrence proportions in the literature range widely and are influenced by follow-up intensity and the definition of failure. Analyses of the Danish registries by Bisgaard et al. [24] and Öberg et al. [12] found no significant difference in second-recurrence rates between revision techniques after a prior laparoendoscopic index repair (Bisgaard: 1/14 vs. 2/73; Öberg: 25/712 [3.5%] vs. 10/169 [5.9%], p = 0.15). In contrast, Herniamed data suggested more second recurrences after repeat laparoendoscopic repair compared with guideline-concordant complementary open repair (8/233 [3.43%] vs. 10/907 [1.1%], p = 0.014); the nationwide rate of second recurrence was 2.72% irrespective of technique [11]. In our cohort, cumulative second recurrences trended higher after ReTEP than after Lichtenstein (~ 31% vs ~ 15%), but the difference did not reach statistical significance. Of note, our prospective clinical and sonographic assessments likely increased detection of oligo- or asymptomatic failures, particularly after ReTEP, and may explain the higher ratesrelative to registry summaries. All ReTEP failures occurred despite surgery by experienced hernia surgeons, arguing against a learning-curve effect. Although underpowered, the finding is clinically relevant and supports guideline recommendations to change the dissection plane at recurrence.

## What these data mean for practice?

### Safety parity, but different risk profiles.

When completed as planned, ReTEP and Lichtenstein show similar overall perioperative morbidity. However, ReTEP entails a higher conversion probability and, compared with complementary TEP in a virgin plane, a higher postoperative hematoma risk—largely attributable to adhesions and hostile scarring in the re-entered posterior field.

### Symptoms matter

CCS captures a mesh- and site-specific symptom burden that appears higher after ReTEP than after complementary Lichtenstein, even when global QoL (COMI-hernia) is similar. These differences are relevant for shared decision-making, especially in patients with pain-sensitive occupations, athletic demands, or low tolerance for foreign-body sensation.

### Recurrence vigilance

The trend toward more second recurrences after ReTEP—while not statistically definitive—should inform counseling and surveillance, particularly when the posterior plane is obviously scarred or prior mesh position/size was suboptimal.

### Surgeon and setting

ReTEP is a high-complexity abdominal wall procedure. Success hinges on exquisite anatomical knowledge, precise preperitoneal technique, and mature extraperitoneal expertise. Centers without sustained volumes and a structured conversion strategy may achieve more predictable outcomes with complementary Lichtenstein in this scenario.

### Strengths and limitations

Strengths include systematic event capture across Clavien–Dindo grades 1–5, granular intraoperative documentation (adhesions, mesh issues), and the dual-instrument PRO approach (CCS and COMI-hernia) combined with clinical and sonographic verification of suspected recurrences.

Limitations center on monocentric design, modest sample sizes, COVID-19-limited follow-up, potential non-response bias in the Lichtenstein PRO subset, and absence of randomization. The recurrence analysis is underpowered and hypothesis-generating.

Future research should adopt harmonized methods and outcomes. Specifically, studies ought to standardize complication definitions and consistently capture all Clavien–Dindo grades to enable valid cross-study comparisons. Prospective, head-to-head trials comparing ReTEP, ReTAPP, and Lichtenstein should use core outcome sets that include the Carolinas Comfort Scale (CCS), COMI-hernia, return-to-function metrics, and costs. Mechanistic endpoints are also needed: quantify total mesh load, characterize mesh–nerve/anatomic interfaces, and evaluate how adhesiolysis strategies influence bleeding and chronic pain. Follow-up should be imaging-enhanced to avoid under-detecting oligo- or asymptomatic failures. Finally, develop and test risk-stratification models—incorporating adhesion severity and prior mesh characteristics—to individualize plane selection and optimize patient-centered outcomes.

## Conclusions

In recurrent inguinal hernia after a prior posterior repair, ReTEP and Lichtenstein have comparable overall perioperative morbidity, but ReTEP carries higher technical friction (adhesions), more conversions, and a greater hematoma formation than posterior repair in a virgin plane. Medium-term CCS outcomes favor Lichtenstein (less foreign-body sensation and pain), whereas global QoL is similar. Second-recurrence proportions trend higher after ReTEP. Until adequately powered prospective trials clarify the durability and PRO balance of redo posterior strategies, plane switching to a complementary anterior repair remains a patient-centered default for the first recurrence after laparoendoscopic primary repair.

## Abbreviation

ASA PS: American Society of Anesthesiologists Physical Status Classification
AWMF: Arbeitsgemeinschaft der Wissenschaftlichen Medizinischen Fachgesellschaften
BfArM: Bundesinstitut für Arzneimittel und Medizinprodukte
BMI: Body-Mass-Index
CAH: Chirurgische Arbeitsgemeinschaft Hernien
CCS: Carolinas Comfort Scale
COMI: Core Outcome Measurement Index
DGAV: Deutsche Gesellschaft für Allgemein- und Viszeralchirurgie
DHG: Deutsche Hernien Gesellschaft
DSGVO: Datenschutz-Grundverordnung
EHS: European Hernia Society
GRADE: Grades of Recommendation, Assessment, Development and Evaluation
IEHS: International Endohernia Society
KDO: Kirchliche Datenschutzverordnung
OCEBM: Oxford Centre for Evidence-Based Medicine
QoL: Quality of Life
STROBE: Strengthening the reporting of observational studies in epidemiology
TAPP: Transabdominal preperitoneal hernia repair
ReTAPP: Redo transabdominal preperitoneal hernia repair
TEP: Totally extraperitoneal hernia repair
ReTEP: Redo total extraperitoneal hernia repair
VAS: Visual Analogue Scale
vs.: Versus
